# Navigating Health Literacy: Comparison of Assessment Tools in a Surgical Safety‐Net Population

**DOI:** 10.1002/wjs.70123

**Published:** 2025-10-22

**Authors:** Mokunfayo O. Fajemisin, Stephanie Martinez Ugarte, Gabrielle E. Hatton, Jackson G. Burns, Lillian S. Kao, Krislynn M. Mueck

**Affiliations:** ^1^ Department of Surgery McGovern Medical School at The University of Texas Health Science Center (UTHealth Houston) Houston Texas USA; ^2^ Department of Surgery Center for Translational Injury Research McGovern Medical School at The University of Texas Health Science Center (UTHealth Houston) Houston Texas USA; ^3^ Center for Surgical Trials and Evidence‐Based Practice McGovern Medical School at The University of Texas Health Science Center (UTHealth Houston) Houston Texas USA; ^4^ The Institute for Clinical Research and Learning Health Care McGovern Medical School at The University of Texas Health Science Center (UTHealth Houston) Houston Texas USA

## Abstract

Health literacy as measured by three validated assessment tools.

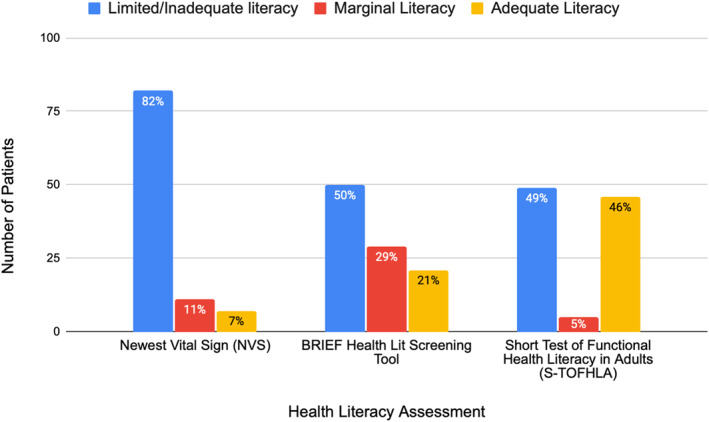

## 1 Introduction

Health literacy (HL) refers to the social and cognitive skills that determine an individual's ability to gain access to, understand, and use information to promote and maintain good health. More than 36% of Americans struggle with low HL, which has been linked to poor health outcomes, increased healthcare expenditures, and reduced access to and quality of care [1]. HL is not routinely assessed clinically and has been understudied in surgical populations [1]. Furthermore, inconsistent use of available validated measurements across studies makes direct comparison challenging [2].

To assess low HL prevalence within our general surgery patients, we developed a prospective survey using 3 validated HL instruments: BRIEF Health Literacy Screening Tool [3], Short Test of Function Health Literacy in Adults (S‐TOFHLA) [4], and Newest Vital Sign (NVS) [5]. The BRIEF, validated in both inpatient and outpatient settings, measures self‐reported health literacy based on patients' confidence levels. The S‐TOFHLA, considered the gold standard for HL assessment, assesses functional health literacy, and the NVS evaluates both reading comprehension and numeracy skills, particularly effective for assessing diverse and low‐income populations; both were validated in outpatient primary care settings. Our goals were to measure the low HL prevalence within our patient population and assess consistency of HL scores among validated HL instruments.

## 2 Methods

We conducted a single‐center prospective survey in adult surgical patients ≥ 18 years at Houston's LBJ Hospital, a busy urban safety‐net hospital, from February 22 to May 15, 2024. We excluded patients with Glasgow Coma score < 15, ICU disposition, and preferred language not English or Spanish. This study was designated quality improvement and thus was exempt from the IRB approval.

Patients' baseline characteristics, demographics, and medical history were collected from electronic medical records. HL scores using 3 validated instruments (see Supporting Information S1: Supplemental Methods) were prospectively collected using patients' preferred languages. All HL instruments were previously validated in both English and Spanish, with a Spanish interpreter involved when necessary. Univariate and multivariable analyses were conducted, and Cramer's *V* coefficients were calculated to compare assessments. Cramer's *V* ranges from 0, indicating no correlation, to 1, indicating perfect correlation.

## 3 Results

**FIGURE 1:**
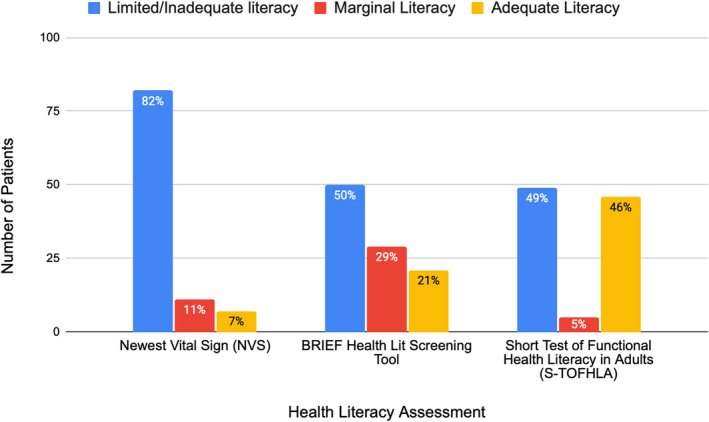
Health literacy as measured by three validated assessment tools.

One‐hundred patients were included; median age was 49 years (IQR 36–62). Most were male, identified as Hispanic or Latino, preferred Spanish language, and had high‐school education (Table 1). A third were uninsured/self‐pay or used discounted medical care (Table 1). Less than half had adequate HL by any method (Figure 1). There was poor correlation among instruments (Cramer's *V* = 0.38), with all tools yielding the same result in only 27% of patients.

**TABLE 1:** Patient characteristics.

	Patients (*n* = 100)
Age, median (IQR)	49 (35.7–62.3)
Sex	
Male (%)	58 (58%)
Female (%)	42 (42%)
Race/Ethnicity	
White	5 (5%)
Black or African American	11 (11%)
Hispanic or Latino	82 (82%)
Other race/Ethnicity	2 (2%)
Preferred language	
English	30 (30%)
Spanish	70 (70%)
Education level	
Professional degree	1 (1%)
Bachelor's degree	4 (4%)
Some college/Associates	7 (7%)
High school	45 (45%)
Middle school	6 (6%)
Elementary school	24 (24%)
No formal education	6 (6%)
Insurance status	
Insured	34 (34%)
Self‐pay/Uninsured	31 (31%)
Medicaid	1 (1%)
Medicare	2 (2%)
Gold‐card (discounted county medical services)	30 (30%)
Other	2 (2%)

### 3.1 NVS

A majority of patients had inadequate (82; 82%) or marginal literacy (11; 11%) by the NVS. On univariate analysis, low HL correlated with education level (*p* < 0.001) but not sex, race/ethnicity, or insurance status/type. On multivariable analysis, no variables were associated with low HL.

### 3.2 BRIEF

With the BRIEF instrument, 50% (*n* = 50) had inadequate literacy whereas 29% (29) had marginal literacy. On univariate analysis, low HL was associated with race/ethnicity (*p* = 0.012) and education level (*p* = 0.007) but not with sex or insurance status/type. On multivariable analysis, low HL correlated with patients lacking formal education (*p* = 0.004) and those listed as other race (*p* = 0.03).

### 3.3 S‐TOFHLA

With the S‐TOFHLA, 49% (49) had inadequate literacy and 5% (5) had marginal literacy. On univariate analysis, low HL was associated with education level (*p* < 0.001) but not race/ethnicity, insurance status/type, or sex. On multivariable analysis, low HL correlated with patients without formal education (*p* = 0.025) or with elementary school level of education (*p* = 0.026).

## 4 Discussion

More than half of surgical patients at our urban safety‐net hospital had low HL regardless of assessment tool used. Our results will be used to inform a mixed‐methods prospective cohort study assessing the relationship between HL and infectious complications after major abdominal surgery as well as a qualitative assessment of how knowledge and HL impact self‐efficacy and wound care. For this study, we will utilize the S‐TOFHLA and BRIEF as they address both functional literacy and patients' perceived literacy.

Ideally communications with patients should be tailored to the HL level. To maximize patient understanding, hospitals with a high proportion of patients with low HL should ensure that communications are provided at sixth grade reading level as recommended by the AMA and NIH [6]. Additionally, careful consideration of which health literacy (HL) tool is used in a given patient population should include an evaluation of the population and setting where the tool was originally validated. Differences in validation criteria among the 3 HL instruments, as well as varying strengths of each tool, may have contributed to lack of consistent results and poor correlation observed among assessments in our study. Finally, social determinants of health greatly influence a patient's HL level and are often correlated; therefore, differences between multivariable and univariate models may be due to effect modification and collinearity. For example, a study comparing the NVS and S‐TOFHLA also noted inconsistencies and found that the NVS was more effective at identifying low HL in younger populations [7]. Therefore, accurate documentation of social determinants of health is important as an adjunct predictor of the HL level.

Targeted interventions should focus on patients who are at increased risk for inadequate HL (e.g., lower educational level and limited insurance coverage). Emphasis should also be placed on interventions designed to address/improve HL and not simply to identify and measure it. Furthermore, as hospitals increasingly seek to address social determinants of health, validity and consistency of measurement tools should be addressed.

## Author Contributions

**Mokunfayo O. Fajemisin:** conceptualization (equal), methodology (equal), investigation (equal), data curation (lead), formal analysis (lead), writing – original draft (lead), writing – review and editing (equal). **Stephanie Martinez Ugarte:** conceptualization (equal), methodology (equal), investigation (equal), data curation (supporting), writing – original draft (supporting), writing – reviewing and editing (equal). **Gabrielle E. Hatton:** conceptualization (equal), methodology (equal), formal analysis (supporting), writing – review and editing (equal). **Jackson G. Burns:** conceptualization (equal), writing – review and editing (equal). **Lillian S. Kao:** conceptualization (equal), methodology (equal), writing – review and editing (equal), supervision (supporting), resources (lead). **Krislynn M. Mueck:** conceptualization (equal), methodology (equal), formal analysis (supporting), supervision (lead), writing – review and editing (equal). We thank Dr. Kimberly Mankiewicz, Center for Translational Injury Research, Department of Surgery, UTHealth Houston, for editing this work.

## Conflicts of Interest

The authors declare no conflicts of interest.

## Supporting information


Supporting Information S1

## Data Availability

Dr. Mokunfayo O. Fajemisin and Dr. Krislynn M. Mueck had full access to all the data in the study and take responsibility for the integrity of the data and accuracy of the data analysis.
