# Supplementary material for: Navigating Health Literacy: Comparison of Assessment Tools in a Surgical Safety‐Net Population
Source: World J Surg. 2025 Oct 22;49(12):3441–3. doi: 10.1002/wjs.70123 (PMC12690011; doi:10.1002/wjs.70123)
Supplement: Supplementary file 1 — Supporting Information S1 [file WJS-49-3441-s001.docx]

**Supplemental Methods**

The BRIEF Health Literacy Screening Tool is a validated 4-item survey where patients self-assess their health literacy using a Likert scale total scores range from 4-20. Inadequate literacy is a score of 4-12, marginal literacy 13-16, and adequate literacy 17-20.^3^ The Short Test of Function Health Literacy in Adults (S-TOFHLA) assesses comprehension using a 36-question survey. Respondents’ complete sentences by selecting the word that best completes each sentence, total scores range from 0-36, a score of 0-16 indicates inadequate literacy, 17-22 indicates marginal literacy, and 23-36 indicates adequate functional literacy.^4^ The Newest Vital Sign (NVS) utilizes a food nutrition label and involves answering 6 questions pertaining to the food label. On the NVS scores of 0-1 indicate a high probability (greater than 50%) of limited literacy, scores of 2-3 indicates possibility of limited literacy, and scores of 4-6 indicates adequate literacy.^5^ For the purposes of this study on the NVS scores of 0-1 indicated low health literacy, and scores of 2-3 indicated marginal literacy.
